# Risk prediction models for macrovascular complications in type 2 diabetes mellitus: from traditional risk scores to multimodal intelligent prediction

**DOI:** 10.3389/fendo.2026.1980631

**Published:** 2026-10-14

**Authors:** Xuedong An, Jingru Cai, Xiaoting Cao, Jinglun Li

**Affiliations:** 1 Guang’anmen Hospital, China Academy of Chinese Medical Sciences, Beijing, China; 2 Endocrinology Department, Liaocheng Hospital of Traditional Chinese Medicine, Liaocheng, China; 3 Department of Traditional Chinese Medicine Rehabilitation, Shiguai District Traditional Chinese Medicine Hospital, Baotou, China

**Keywords:** machine learning, macrovascular complications, precision medicine, risk prediction, type 2 diabetes mellitus

## Abstract

Macrovascular complications are the leading cause of morbidity and mortality in patients with type 2 diabetes mellitus (T2DM). Early risk identification and accurate risk stratification are essential for both primary and secondary cardiovascular prevention. With the rapid development of electronic health records, medical imaging, multi-omics technologies, and artificial intelligence (AI), risk prediction models for diabetic macrovascular complications have evolved from traditional statistical approaches to machine learning-, deep learning-, and multimodal AI-driven models. However, despite substantial improvements in predictive performance, current models remain limited by heterogeneous target populations, inconsistent outcome definitions, insufficient external validation, limited clinical interpretability, and inadequate real-world implementation, thereby restricting their clinical translation. This review summarizes the evolution, data sources, modeling methodologies, clinical applications, and future perspectives of risk prediction models for macrovascular complications in T2DM. We first describe the transition from conventional cardiovascular risk scores, including the Framingham Risk Score, to diabetes-specific models such as the UKPDS Risk Engine, ADVANCE, RECODe, and SCORE2-Diabetes, followed by the emergence of AI-based prediction models. We then review the major data sources used in contemporary prediction models, including clinical characteristics, laboratory biomarkers, vascular function and imaging, multi-omics profiles, and wearable device-derived data. Furthermore, we compare the strengths and limitations of traditional statistical methods, machine learning, deep learning, and multitask learning with respect to predictive performance, interpretability, and clinical applicability, and discuss the major challenges related to study design, external validation, data quality, algorithmic bias, and real-world implementation. Finally, we propose that future risk prediction models for diabetic macrovascular complications will evolve from single-source data to multimodal data integration, from static risk estimation to dynamic risk trajectories, from single outcomes to multiple interconnected cardiovascular outcomes, from prognosis prediction to individualized treatment benefit prediction, and from standalone algorithms to continuously learning clinical decision support systems. Emerging technologies, including foundation models, large language models, digital twins, causal inference, and continuous learning frameworks, are expected to drive the transition from Risk Prediction to Clinical Decision and ultimately Patient Benefit, thereby advancing precision prevention and individualized management of diabetes.

## 1 Introduction

Type 2 diabetes mellitus (T2DM) is a chronic systemic metabolic disorder characterized by persistent hyperglycemia that affects multiple organ systems and contributes substantially to global morbidity and mortality (1). Despite remarkable advances in glucose-lowering therapies over the past decades, diabetes-related macrovascular and microvascular complications remain the major determinants of long-term prognosis, indicating that intensive glycemic control alone is insufficient to fully prevent the development and progression of vascular damage (2, 3). Macrovascular complications—including atherosclerotic cardiovascular disease (ASCVD), coronary artery disease (CAD), myocardial infarction (MI), ischemic stroke, heart failure (HF), and peripheral artery disease (PAD)—account for approximately 70%–80% of deaths among patients with T2DM, with ASCVD remaining the leading cause of mortality (4, 5). Therefore, early identification of high-risk individuals and accurate cardiovascular risk stratification are essential for improving long-term clinical outcomes.

The development of diabetic macrovascular complications is driven by the cumulative effects of chronic metabolic disturbances and multiple interacting risk factors, involving insulin resistance, chronic inflammation, oxidative stress, dyslipidemia, endothelial dysfunction, and atherosclerosis. Consequently, cardiovascular risk in patients with T2DM is inherently multifactorial, dynamic, and highly heterogeneous. Current clinical risk assessment primarily relies on conventional risk scores based on traditional cardiovascular risk factors, including age, sex, blood pressure, lipid profiles, glycemic status, smoking, and body mass index (BMI), which provide an important foundation for population-level risk stratification (6–12). However, these models are largely based on conventional statistical approaches and have limited ability to capture complex nonlinear interactions, temporal changes in risk factors, or integrate emerging data sources such as biomarkers, medical imaging, multi-omics, and longitudinal real-world health data. As precision medicine advances, conventional risk prediction models face increasing challenges in both predictive performance and generalizability (13, 14).

Recent advances in artificial intelligence (AI), machine learning (ML), and deep learning (DL) have provided new opportunities for cardiovascular risk prediction in diabetes. Compared with traditional statistical models, AI-based approaches can integrate heterogeneous data from multiple sources—including clinical characteristics, laboratory biomarkers, medical imaging, wearable devices, and multi-omics profiles—and automatically learn complex nonlinear relationships, demonstrating promising predictive performance across oncology, cardiovascular disease, chronic kidney disease, and diabetic complications (15–17). Meanwhile, risk prediction is evolving from static, single-time-point assessment toward dynamic prediction driven by multimodal data integration, multitask learning, foundation models, and digital twins, creating new opportunities for individualized risk assessment and clinical decision support.

Despite these advances, current prediction models for diabetic macrovascular complications still face several important challenges, including substantial model heterogeneity, insufficient external validation, limited capability for dynamic risk prediction, inadequate interpretability and fairness, and poor real-world implementation, all of which hinder their translation into routine clinical practice (18–21). Moreover, existing reviews have primarily focused on algorithmic development or comparisons of predictive performance, while lacking a comprehensive overview of the evolution of prediction models, changes in data sources, multimodal AI-driven prediction, implementation pathways, and future applications in precision medicine. Therefore, this review provides a comprehensive overview of the evolution of risk prediction models for diabetic macrovascular complications, tracing their development from traditional risk scores to machine learning, deep learning, and multimodal AI-based approaches. We summarize the major data sources, modeling strategies, performance evaluation methods, clinical applications, and implementation challenges, and further discuss emerging directions—including foundation models, digital twins, continual learning, and precision medicine—to illustrate the transition of risk prediction from Prediction to Decision and ultimately Benefit, with the goal of advancing precision screening, individualized intervention, and intelligent management of diabetic macrovascular complications.

## 2 Evolution of risk prediction models for diabetic macrovascular complications

The evolution of risk prediction models for diabetic macrovascular complications reflects a continuous shift in the paradigm of cardiovascular risk assessment—from population-based risk scores to diabetes-specific models, and more recently to AI-driven multimodal prediction. The Framingham Risk Score laid the foundation for modern cardiovascular risk prediction by estimating the risk of coronary heart disease using traditional risk factors such as age, sex, smoking status, blood pressure, and lipid levels, thereby providing an important tool for primary prevention (22). However, because it was developed in the general population, it does not incorporate diabetes-specific characteristics, such as disease duration and glycemic control, and therefore tends to overestimate cardiovascular risk and demonstrates suboptimal calibration in patients with diabetes (23) (**Figure 1**).

**Figure 1:**
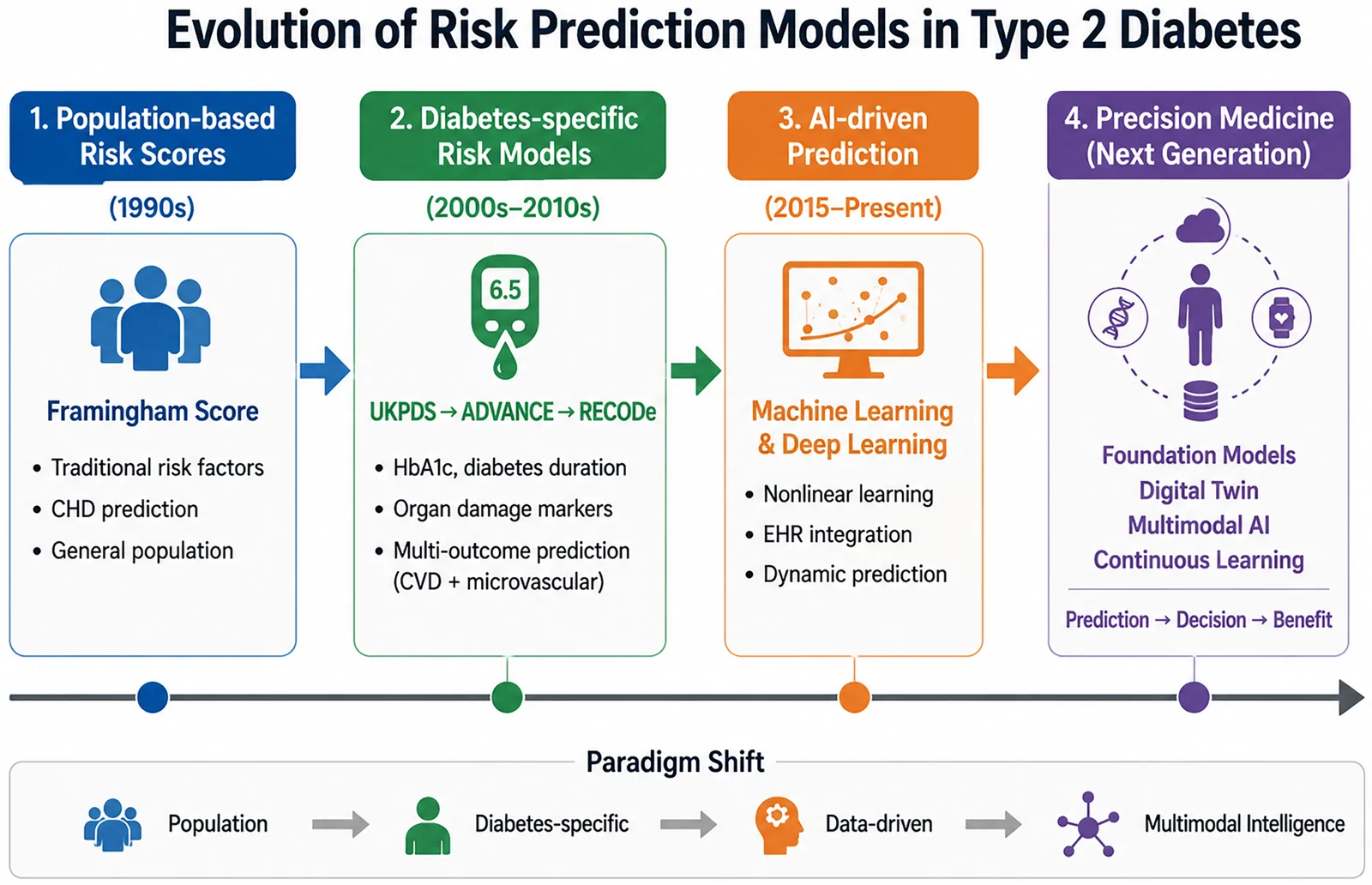
Evolution of risk prediction models for diabetic macrovascular complications.

To enable individualized cardiovascular risk assessment in diabetes, the UKPDS Risk Engine was the first disease-specific prediction model to incorporate diabetes-related variables, including glycated hemoglobin (HbA1c) and diabetes duration (24). Subsequently, the ADVANCE Risk Model further improved prediction by integrating markers of target-organ damage, such as the urinary albumin-to-creatinine ratio, diabetic retinopathy, atrial fibrillation, and non-high-density lipoprotein cholesterol (non-HDL-C) (25). Based on data from large clinical trials, the RECODe model expanded prediction beyond cardiovascular events to include microvascular complications and demonstrated superior discrimination and calibration compared with traditional models such as the UKPDS Risk Engine (26). This stage marked the transition from population-based to diabetes-specific prediction models, from conventional cardiovascular risk factors to markers of organ damage, and from single-outcome prediction to comprehensive prediction of multiple diabetes-related complications.

More recently, SCORE2-Diabetes was developed to provide contemporary 10-year cardiovascular risk estimation specifically for individuals with T2DM (27). Building on the SCORE2 framework, the model incorporates diabetes-specific variables, including age at diabetes diagnosis, HbA1c, and eGFR, in addition to conventional cardiovascular risk factors (27). One of its major strengths is the use of contemporary European cohorts, competing-risk adjustment, and regional recalibration according to different baseline cardiovascular risk levels. Compared with general-population risk equations, SCORE2-Diabetes provides a more diabetes-specific framework for primary cardiovascular prevention. However, its applicability is mainly established in European populations without established cardiovascular disease, and its transportability to non-European populations, secondary prevention settings, and patients with advanced target-organ damage requires further validation (27, 28).

With the rapid accumulation of electronic health records (EHRs) and real-world data, conventional regression-based models, such as the Cox proportional hazards model, have become increasingly limited in handling high-dimensional data, nonlinear relationships, and complex feature interactions. Consequently, risk prediction has gradually shifted from hypothesis-driven statistical modeling to data-driven approaches (29). ML algorithms—including random forests, gradient boosting decision trees, LightGBM, and CatBoost—can automatically learn complex patterns from heterogeneous data and have consistently demonstrated superior predictive performance compared with traditional statistical models in multiple studies (30–33). Furthermore, DL enables dynamic risk prediction by leveraging longitudinal and temporal health data. For example, Lee et al. developed a GRU-ODE-Bayes model that outperformed conventional logistic regression and XGBoost models (AUROC = 0.812), highlighting the potential advantages of dynamic prediction (34).

More recently, foundation models and digital twins have further advanced cardiovascular risk prediction into the era of precision medicine. Through large-scale pretraining on EHR data, foundation models can learn generalizable patient representations, support multitask prediction, and facilitate cross-institutional transfer learning, providing a new framework for clinical risk prediction (35). In parallel, digital twins continuously integrate data from continuous glucose monitoring, laboratory tests, lifestyle factors, and therapeutic interventions to construct dynamic virtual representations of individual patients. These models enable simulation of disease progression and personalized intervention strategies and have shown considerable promise for precision diabetes management (36).

Overall, the evolution of risk prediction models for diabetic macrovascular complications can be summarized into four major transitions: from population-based models to diabetes-specific models, from traditional statistical methods to data-driven approaches, from static to dynamic prediction, and from single-outcome prediction to multimodal intelligent prediction. Looking forward, foundation models, digital twins, and multimodal AI are expected to further transform risk prediction from Prediction to Decision and ultimately Benefit, enabling precision risk management throughout the entire disease course of patients with diabetes.

## 3 Data sources for risk prediction models of diabetic macrovascular complications

### 3.1 Clinical characteristics

Clinical characteristics constitute the most fundamental and widely used data source for risk prediction models of diabetic macrovascular complications because of their accessibility, low cost, and high degree of standardization. Demographic characteristics, diabetes-related clinical history, and traditional cardiovascular risk factors form the core variables of nearly all prediction models. From the Framingham Risk Score and the UKPDS Risk Engine to the ADVANCE, RECODe, and SCORE2-Diabetes models, diabetes-specific variables have been progressively incorporated into conventional clinical risk factors, leading to continuous improvements in predictive performance.

Demographic variables primarily include age, sex, ethnicity, and socioeconomic factors. Among these, age and sex are common predictors shared by the Framingham, UKPDS, ADVANCE, and RECODe models (37, 38). More recently, social determinants of health, including ethnicity, socioeconomic status, and educational attainment, have been increasingly incorporated into cardiovascular risk assessment to improve risk stratification across diverse populations. Diabetes-specific variables—including diabetes duration, age at diagnosis, HbA1c, glucose-lowering therapy, insulin use, and diabetes subtype—represent key components of diabetes-specific prediction models. Diabetes duration and HbA1c have been widely adopted in the UKPDS, ADVANCE, and RECODe models, whereas dynamic glycemic metrics, such as time-weighted HbA1c, HbA1c variability, and glycemic burden, provide a more comprehensive assessment of long-term glycemic exposure and have emerged as important directions for model refinement. The SCORE2-Diabetes model further incorporates age at diabetes diagnosis, HbA1c, and estimated glomerular filtration rate (eGFR), thereby further improving cardiovascular risk prediction (27).

Traditional ASCVD risk factors—including obesity, blood pressure, lipid profiles, smoking, and chronic kidney disease—remain the common foundation of the Framingham, UKPDS, ADVANCE, RECODe, and SCORE2-Diabetes models (39, 40). In recent years, risk prediction has gradually shifted from individual risk factors toward comprehensive assessment of health behaviors. Composite metrics such as the Lifestyle Score, Healthy Behavior Score, and the Life’s Essential 8 integrate information on smoking, diet, physical activity, sleep, and metabolic health, providing a more comprehensive representation of cumulative lifetime risk exposure. These behavioral metrics have been increasingly incorporated into cardiovascular risk prediction models for patients with diabetes to further improve risk stratification (41–44).

### 3.2 Laboratory biomarkers

Laboratory biomarkers represent one of the most important data sources for risk prediction models of diabetic macrovascular complications because they are objective, readily available, and suitable for repeated measurements. They reflect multiple pathophysiological processes, including glucose metabolism, lipid metabolism, renal dysfunction, myocardial injury, inflammation, and insulin resistance. Conventional laboratory biomarkers, including HbA1c, lipid profiles, eeGFR, and urinary albumin, have been widely incorporated into established prediction models such as the UKPDS Risk Engine, ADVANCE Risk Model, RECODe Model, and SCORE2-Diabetes. In contrast, N-terminal pro-B-type natriuretic peptide (NT-proBNP), high-sensitivity cardiac troponin (hs-cTn), the triglyceride-glucose (TyG) index, and inflammatory biomarkers have mainly been investigated as incremental predictors to further optimize cardiovascular risk stratification (45).

HbA1c is the most established biomarker of glycemic control and has been widely incorporated into classical prediction models, including the UKPDS Risk Engine, ADVANCE Risk Model, RECODe Model, and SCORE2-Diabetes. More recently, dynamic glycemic metrics, including time-weighted HbA1c, HbA1c variability, glycemic burden, and continuous glucose monitoring (CGM)-derived parameters such as time in range (TIR), time above range (TAR), and time below range (TBR), have been shown to more accurately reflect long-term glycemic exposure and have gradually become important directions for model optimization (46). Conventional lipid biomarkers, including total cholesterol (TC), low-density lipoprotein cholesterol (LDL-C), high-density lipoprotein cholesterol (HDL-C), and triglycerides (TG), remain the most frequently used lipid variables in cardiovascular risk prediction models (47–51). In recent years, emerging lipid biomarkers, including apolipoprotein B (ApoB), lipoprotein(a) [Lp(a)], small dense LDL (sdLDL), remnant cholesterol, and lipoprotein-associated phospholipase A2 (Lp-PLA2), together with renal biomarkers such as eGFR, urinary albumin-to-creatinine ratio (UACR), blood urea nitrogen-to-albumin ratio (BAR), and cystatin C, have demonstrated additional value for cardiovascular risk stratification. Among these, eGFR and UACR have already been incorporated into established prediction models, including ADVANCE, RECODe Model, and SCORE2-Diabetes (45, 52).

Cardiac biomarkers, including NT-proBNP, B-type natriuretic peptide (BNP), and high-sensitivity cardiac troponin, reflect myocardial injury and can further improve the prediction of heart failure, cardiovascular mortality, and composite cardiovascular events when added to conventional risk models. Novel biomarkers, including growth differentiation factor-15 (GDF-15), galectin-3, soluble suppression of tumorigenicity-2 (sST2), and mid-regional pro-adrenomedullin (MR-proADM), are currently being investigated primarily for model optimization (53–58). Inflammatory biomarkers, including C-reactive protein (CRP), interleukin-6 (IL-6), tumor necrosis factor-α (TNF-α), as well as inflammation-derived indices such as the neutrophil-to-lymphocyte ratio (NLR), platelet-to-lymphocyte ratio (PLR), systemic immune-inflammation index (SII), and systemic inflammation response index (SIRI), have all been shown to be closely associated with cardiovascular events in patients with diabetes. Compared with individual inflammatory markers, multi-protein inflammatory panels appear to have greater potential for improving cardiovascular risk prediction (59–62).

In addition, markers of insulin resistance, including the TyG index, TyG-BMI, TG/HDL-C ratio, and homeostasis model assessment of insulin resistance (HOMA-IR), together with oxidative stress biomarkers such as oxidized LDL (ox-LDL), advanced glycation end products (AGEs), uric acid, and ferritin, have increasingly become research hotspots in cardiovascular risk prediction. However, their incremental clinical value beyond established prediction models remains to be further validated (63–66).

Overall, laboratory biomarkers have evolved from conventional biochemical measurements toward a broader spectrum encompassing organ damage biomarkers, dynamic metabolic indices, and emerging molecular biomarkers. Among these, HbA1c, lipid profiles, eGFR, and UACR constitute the core laboratory variables incorporated into established prediction models. NT-proBNP and high-sensitivity cardiac troponin have demonstrated substantial incremental predictive value, whereas biomarkers such as the TyG index, HbA1c variability, ApoB, Lp(a), SIRI, BAR, and GDF-15 remain in the early stage of clinical translation. Future studies should focus on evaluating the incremental predictive value of these biomarkers beyond existing prediction models and facilitate the transition from single-point laboratory measurements toward longitudinal trajectory-based biomarker assessment, thereby enabling more accurate and dynamic cardiovascular risk prediction in patients with diabetes.

### 3.3 Vascular function and imaging biomarkers

Vascular function and cardiovascular imaging biomarkers directly reflect endothelial dysfunction, arterial stiffness, subclinical atherosclerosis, and myocardial injury, and have emerged as important data sources for risk prediction models of diabetic macrovascular complications in recent years. Compared with traditional cardiovascular risk factors, these biomarkers are more closely related to the underlying vascular pathology, improve risk reclassification, and can be integrated with clinical characteristics and laboratory biomarkers to develop novel prediction models. However, established risk prediction models, including the UKPDS Risk Engine, ADVANCE Risk Model, RECODe Model, and SCORE2-Diabetes, have not yet routinely incorporated these vascular function and imaging parameters.

Vascular function indices, including pulse wave velocity (PWV), ankle–brachial index (ABI), flow-mediated dilation (FMD), cardio-ankle vascular index (CAVI), and reactive hyperemia index (RHI), reflect arterial stiffness and endothelial function, respectively. Among these, PWV and ABI have the strongest evidence base and have been shown to improve cardiovascular risk stratification when added to conventional prediction models. In contrast, FMD is more suitable for evaluating early endothelial dysfunction but has not yet been widely incorporated into established risk prediction models (67–71). Carotid ultrasonography enables the assessment of carotid intima–media thickness (cIMT), carotid plaques, and plaque burden, with plaque burden demonstrating greater predictive value than cIMT alone. In recent years, machine learning models integrating carotid ultrasound findings with clinical and metabolic variables have outperformed conventional cardiovascular risk scores, suggesting that combining imaging and clinical data may improve early identification of individuals at high cardiovascular risk (72).

Coronary artery calcium (CAC) is currently one of the most extensively validated imaging biomarkers for cardiovascular risk stratification and has been shown to further improve risk assessment beyond traditional prediction models (73). Coronary computed tomography angiography (CCTA) and computed tomography-derived fractional flow reserve (CT-FFR), by integrating plaque characteristics with functional hemodynamic information, can further enhance the prediction of major adverse cardiovascular events. Emerging imaging biomarkers, such as epicardial adipose tissue, are primarily being investigated for prediction model optimization (74–76). Cardiac magnetic resonance (CMR) enables comprehensive evaluation of myocardial ischemia, myocardial scar, and diffuse myocardial fibrosis, providing unique advantages for detecting subclinical myocardial injury. However, owing to its relatively high cost and limited accessibility, CMR is currently used mainly for the evaluation of high-risk patients (77).

More recently, AI has enabled the extraction of high-dimensional imaging features from ultrasound, computed tomography (CT), and magnetic resonance imaging (MRI), facilitating the development of multimodal prediction models through integration with clinical characteristics and laboratory biomarkers. These AI-based multimodal models have demonstrated superior predictive performance compared with conventional cardiovascular risk scores, although evidence regarding external validation and clinical implementation remains limited (72, 75).

Overall, vascular function and imaging biomarkers have evolved from simple structural assessments to comprehensive indicators of vascular function, plaque characteristics, and myocardial injury. Among these biomarkers, PWV, ABI, carotid plaque burden, and CAC have the most robust evidence supporting their clinical application, whereas CCTA, CT-FFR, CMR, and AI-enabled multimodal imaging represent important future directions in cardiovascular risk prediction. Future studies should focus on evaluating the incremental predictive value of these biomarkers beyond existing prediction models and promoting their clinical implementation through multicenter external validation and assessments of clinical net benefit.

### 3.4 Multi-omics biomarkers

With the rapid development of high-throughput sequencing and multi-omics technologies, risk prediction for diabetic macrovascular complications has gradually evolved from conventional clinical indicators toward molecular-level characterization. Multi-omics data, including genomics, proteomics, metabolomics, lipidomics, gut microbiomics, and single-cell omics, provide insights into the biological mechanisms underlying disease onset and progression from multiple dimensions, including genetic susceptibility, molecular alterations, and metabolic reprogramming. Increasingly, these molecular data are being integrated with conventional risk prediction models to improve cardiovascular risk stratification.

Genome-wide association studies (GWAS) and polygenic risk scores (PRS) enable the assessment of individual genetic susceptibility. Large multi-ancestry studies have identified hundreds of genetic loci associated with T2DM and its vascular complications. PRS has been shown to be associated with the risks of coronary heart disease and peripheral artery disease (78). However, its incremental predictive value beyond conventional risk factors remains relatively limited, making it more suitable as a complementary indicator for long-term baseline cardiovascular risk assessment rather than as an independent prediction tool (79). Among the available multi-omics approaches, proteomics has achieved the greatest degree of clinical translation. Proteomic biomarkers have been shown to significantly improve the predictive performance of conventional risk models and have already been incorporated into the optimization of models such as SCORE2-Diabetes. Metabolomics and lipidomics provide comprehensive information on metabolic abnormalities and atherosclerosis-related pathways. Key metabolites and lipid biomarkers have demonstrated additional predictive value for cardiovascular risk and complement proteomic data by capturing distinct biological processes involved in disease progression (79–84).

The gut microbiome has emerged as another promising source of cardiovascular biomarkers. Through metabolites such as trimethylamine N-oxide (TMAO), short-chain fatty acids (SCFAs), and bile acids, the gut microbiota modulates inflammation, lipid metabolism, and vascular function and has been closely associated with diabetic macrovascular complications. Nevertheless, microbiome-derived biomarkers have not yet been widely incorporated into established cardiovascular risk prediction models (85–87). Single-cell omics enables the characterization of dynamic changes in vascular-associated cell populations and provides a powerful approach for identifying novel molecular biomarkers and therapeutic targets. However, its application in cardiovascular risk prediction remains at an exploratory stage.

Overall, multi-omics technologies have expanded cardiovascular risk prediction beyond conventional clinical risk factors toward molecular phenotyping. Among these technologies, proteomics and metabolomics have demonstrated the greatest potential for clinical translation, whereas genomics is primarily used for assessing inherited cardiovascular risk. Gut microbiomics and single-cell omics represent important future directions for biomarker discovery and precision risk prediction. In the future, the integration of multi-omics data with artificial intelligence, multimodal data fusion, and longitudinal dynamic monitoring is expected to further advance risk prediction for diabetic macrovascular complications toward the era of precision medicine.

### 3.5 Wearable devices

Wearable devices and mobile health (mHealth) technologies enable the continuous collection of dynamic physiological data, including glucose levels, heart rate, heart rate variability (HRV), sleep patterns, and physical activity. These technologies provide longitudinal time-series data that are difficult to obtain through conventional outpatient assessments and have emerged as an important data source for digital risk prediction of diabetic macrovascular complications. Among currently available wearable technologies, CGM has the strongest evidence base, whereas smart wearable devices and digital biomarkers remain at an exploratory stage.

CGM generates a range of glycemic metrics, including TIR, TAR, TBR, and glycemic variability, which more comprehensively reflect long-term glucose fluctuations than HbA1c. These metrics have been shown to be closely associated with diabetic vascular complications and clinical prognosis (88, 89). In recent years, CGM-based research has gradually shifted from analyses based on individual glycemic metrics to time-series modeling. Foundation models such as GluFormer leverage digital representations derived from CGM data to further improve the prediction of diabetes-related outcomes and cardiovascular mortality (90).

Smartwatches and wearable electrocardiographic devices enable continuous monitoring of digital biomarkers, including HRV, sleep duration and quality, step count, and physical activity, all of which have been associated with cardiovascular risk in individuals with diabetes. However, the independent predictive value of these wearable-derived digital biomarkers requires further validation (91). In the future, multimodal prediction models integrating CGM-derived metrics, HRV, sleep characteristics, physical activity, and conventional clinical variables are expected to facilitate personalized and dynamic cardiovascular risk prediction. In addition, mHealth applications and digital disease management platforms will provide novel real-world data sources for continuous risk assessment and longitudinal disease monitoring (91–94).

Overall, wearable technologies are transforming risk prediction for diabetic macrovascular complications from single-point static measurements to continuous dynamic monitoring. Among wearable-derived biomarkers, CGM and its derived glycemic metrics currently have the most robust evidence supporting their clinical utility, whereas digital biomarkers, multimodal data integration, and artificial intelligence-driven analytics represent the major future directions for precision cardiovascular risk prediction in patients with diabetes.

## 4 Current status of risk prediction models for diabetic macrovascular complications

Risk prediction models for diabetic macrovascular complications have evolved progressively from traditional statistical models to machine learning, deep learning, and multi-task learning approaches. Each methodology possesses distinct strengths in variable processing, nonlinear relationship modeling, interpretability, and clinical applicability, collectively driving the transition of cardiovascular risk prediction from static assessments based on a limited number of conventional risk factors toward multimodal, dynamic, and precision prediction.

Traditional statistical models form the foundation of cardiovascular risk prediction in diabetes and remain the most widely used approaches in clinical practice. The major methodologies include logistic regression, Cox proportional hazards models, Fine–Gray competing risk models, risk scores, and nomograms. Logistic regression is primarily used to predict the occurrence of events at a predefined time point, whereas Cox proportional hazards models are applicable to longitudinal follow-up data and continue to serve as the predominant method for developing cardiovascular risk prediction models. In contrast, Fine–Gray competing risk models are particularly suitable for long-term follow-up studies in which competing events may influence the occurrence of the primary outcome (95–98). Based on these statistical approaches, several classical cardiovascular risk prediction models have been developed, including the Framingham Risk Score, UKPDS Risk Engine, ADVANCE Risk Model, RECODe Model, the Swedish National Diabetes Register (NDR) Risk Model, and the Pooled Cohort Equations (PCE) (24–26, 99–107). These models are characterized by relatively simple structures, strong interpretability, and ease of clinical implementation. Their externally validated C-statistics generally range from 0.66 to 0.79, with RECODe demonstrating comparatively superior overall predictive performance, whereas the Framingham Risk Score and PCE exhibit relatively limited predictive accuracy in populations with diabetes. Overall, traditional statistical models remain the cornerstone of cardiovascular risk assessment in clinical practice; however, their ability to model high-dimensional data, complex nonlinear relationships, and interactions among variables is inherently limited.

With the rapid accumulation of electronic health records (EHRs), multi-omics data, and medical imaging datasets, ML has emerged as an increasingly important approach for predicting diabetic macrovascular complications. Compared with traditional statistical models, ML algorithms can automatically capture complex nonlinear relationships among variables and are therefore better suited for analyzing high-dimensional and multimodal data (108–110). Commonly used algorithms include random forests, support vector machines (SVMs), Extreme Gradient Boosting (XGBoost), Light Gradient Boosting Machine (LightGBM), CatBoost, artificial neural networks (ANNs), and Bayesian networks (111–114). Among these, gradient boosting tree algorithms have demonstrated excellent predictive performance and computational efficiency, making them one of the most widely adopted approaches in recent years. Artificial neural networks possess strong nonlinear modeling capabilities, with some studies reporting areas under the receiver operating characteristic curve (AUCs) as high as 0.91. Bayesian networks combine favorable predictive performance with inherent interpretability, thereby providing both risk estimation and probabilistic reasoning. More recently, ML has been increasingly applied to patient phenotyping, prediction of treatment benefit, and precision medicine, including the identification of patients most likely to benefit from intensive glucose-lowering therapy and the characterization of distinct coronary plaque phenotypes (115–118). Overall, although most studies have reported superior predictive performance of ML models compared with traditional statistical approaches, the performance gap becomes less pronounced when studies employ more rigorous methodological designs and comprehensive external validation (119).

DL has further expanded the application of machine learning to longitudinal data, medical imaging, and multimodal data integration. In recent years, convolutional neural networks (CNNs), recurrent neural networks (RNNs), Transformers, graph neural networks (GNNs), and deep survival learning models, such as DeepSurv and DeepHit, have been increasingly applied to cardiovascular risk prediction in patients with diabetes. CNNs are primarily used for medical image analysis, whereas RNNs and Transformer architectures are particularly suitable for modeling longitudinal electronic health records and continuously monitored physiological data. GNNs enable the modeling of patient similarity networks and complex multi-omics interactions, while deep survival learning models directly estimate time-to-event risks over different follow-up periods (120, 121). Meanwhile, advances in explainable artificial intelligence (XAI) have further improved the clinical applicability of deep learning models. SHapley Additive exPlanations (SHAP) quantify the contribution of individual variables to model predictions, whereas Gradient-weighted Class Activation Mapping (Grad-CAM) identifies key regions within medical images that drive model decisions, thereby providing biologically meaningful interpretations of prediction results. Overall, deep learning exhibits substantial advantages in complex pattern recognition and multimodal data fusion. Nevertheless, challenges remain, including the need for large training datasets, limited model interpretability, and insufficient evidence supporting routine clinical implementation.

In recent years, cardiovascular risk prediction has gradually evolved from predicting a single endpoint to simultaneously predicting multiple clinically related outcomes, making multi-task learning (MTL) an important direction in artificial intelligence–based risk prediction. Unlike conventional approaches that develop separate prediction models for ASCVD, heart failure, stroke, or all-cause mortality, MTL shares information across related prediction tasks and simultaneously predicts multiple clinically relevant outcomes, thereby improving model generalizability and predictive efficiency. Currently, MTL is increasingly being integrated with Transformer architectures, graph neural networks, and deep survival learning models to simultaneously predict multiple cardiovascular and cardiometabolic outcomes, including ASCVD, heart failure, stroke, mortality, and cardiovascular–kidney–metabolic (CKM) syndrome. These models also facilitate the integration of clinical characteristics, laboratory biomarkers, medical imaging, and multi-omics data, enabling comprehensive multimodal and dynamic cardiovascular risk assessment (122–124). Although MTL remains in the early stage of clinical translation, the continued expansion of large-scale electronic health record databases and the emergence of foundation models are expected to make multi-task learning one of the most promising directions for the next generation of risk prediction models for diabetic macrovascular complications.

Overall, the development of risk prediction models for diabetic macrovascular complications has evolved through four major stages: traditional statistical models, machine learning, deep learning, and multi-task learning. Traditional statistical models remain the cornerstone of clinical risk assessment, whereas machine learning and deep learning have substantially enhanced the ability to analyze high-dimensional and complex data. More recently, multi-task learning has further advanced cardiovascular risk prediction by shifting the paradigm from single-outcome prediction to simultaneous prediction of multiple clinically related outcomes, from static risk assessment to dynamic risk prediction, and from unimodal analysis to multimodal data integration. Collectively, these advances provide a new technological framework for precision risk stratification and individualized clinical decision-making.

## 5 Comparative evaluation of major risk prediction models for diabetic macrovascular complications

Numerous cardiovascular risk prediction models have been developed for patients with T2DM; however, direct comparison remains challenging because these models differ substantially in target populations, predicted outcomes, prediction horizons, predictor variables, and validation settings. Therefore, rather than identifying a universally superior model, comparative evaluation should focus on the clinical context in which each model was developed, its discrimination and calibration, external validation, interpretability, and feasibility of implementation. **Table 1** summarizes the major characteristics, strengths, limitations, and potential clinical applications of representative risk prediction models.

**Table 1:** Comparative characteristics, performance, strengths, limitations, and clinical applicability of representative cardiovascular risk prediction models in T2DM.

Model	Development population	Main predicted outcome	Prediction horizon	Key predictors	Method	Reported performance*	Main strengths	Main limitations	Potential clinical applicability
Framingham Risk Score (23)	General population; Framingham Heart Study	CHD/CVD	Usually 10 years	Age, sex, smoking, SBP, cholesterol	Cox regression	Modest performance in contemporary T2DM cohorts; C-statistics approximately 0.57–0.71 in ADVANCE validation	Simple; widely known; easy to implement; requires routinely available variables	Not diabetes-specific; does not incorporate diabetes duration or glycemic control; may substantially overestimate risk in contemporary T2DM populations	General cardiovascular risk assessment when diabetes-specific tools are unavailable
UKPDS Risk Engine (24)	Patients with newly diagnosed T2DM in UKPDS	CHD; stroke	Up to 10 years	Age, sex, ethnicity, smoking, diabetes duration, HbA1c, SBP, TC/HDL-C	Parametric survival models	Moderate discrimination; performance deteriorates in contemporary external cohorts	Diabetes-specific; incorporates HbA1c and diabetes duration; historically important	Developed from an older treatment era; calibration drift; may overestimate current cardiovascular risk	Diabetes-specific risk assessment, particularly of historical and research value
ADVANCE Risk Model (25)	T2DM participants in ADVANCE	Major CVD	Approximately 4–5 years	Age at diagnosis, diabetes duration, sex, pulse pressure, treated hypertension, AF, retinopathy, HbA1c, UACR, non-HDL-C	Cox regression	C-statistic ≈0.70 internally and ≈0.69 externally	Incorporates diabetes-specific factors and markers of target-organ damage; uses routinely available variables	Developed largely from a clinical-trial population; limited prediction horizon; requires recalibration in other populations	Cardiovascular risk stratification in patients with established T2DM
Swedish NDR Risk Model (103)	Large real-world Swedish T2DM registry	Fatal/nonfatal CVD	5 years	Age at diabetes onset, duration, sex, HbA1c, SBP, BMI, smoking, TC/HDL-C, albuminuria, AF, previous CVD	Cox regression	C-statistic ≈0.72 in validation cohort	Derived from routine-care data; incorporates albuminuria and previous CVD; good clinical accessibility	Primarily developed in a Swedish population; generalizability to other ethnic and healthcare settings requires validation	Real-world cardiovascular risk stratification and routine diabetes care
RECODe (26)	ACCORD participants; externally tested in Look AHEAD/DPPOS	CVD and multiple diabetes complications	Typically 10 years	Demographics, diabetes duration, HbA1c, BP, lipid profile, smoking, renal markers and treatment variables	Regression-based risk equations	Good discrimination and calibration in external validation; generally superior to older diabetes-specific equations	Predicts multiple macrovascular and microvascular complications; developed using contemporary trial data; externally validated	Greater model complexity; treatment patterns and baseline risk continue to evolve; recalibration may be required	Comprehensive prediction of multiple diabetes-related complications
SCORE2-Diabetes (27)	229,460 European individuals with T2DM without previous CVD; external validation in 217,036 individuals	Fatal and nonfatal CVD	10 years	Age, smoking, SBP, total cholesterol, HDL-C plus age at diabetes diagnosis, HbA1c and eGFR	Sex-specific competing-risk-adjusted models with regional recalibration	Improved discrimination over SCORE2; C-index increment 0.009–0.031; satisfactory regional calibration	Contemporary large-scale derivation and validation; diabetes-specific variables; competing-risk adjustment; recalibrated across four European risk regions	Primarily validated in European primary-prevention populations; limited applicability to established CVD, severe target-organ damage and non-European populations without further validation	Contemporary primary cardiovascular prevention and risk stratification in European patients with T2DM

AF, atrial fibrillation; CVD, cardiovascular disease; CHD, coronary heart disease; eGFR, estimated glomerular filtration rate; HbA1c, glycated hemoglobin; NDR, National Diabetes Register; SBP, systolic blood pressure; UACR, urinary albumin-to-creatinine ratio.

Direct comparison of discrimination metrics across models should be interpreted cautiously because of differences in development populations, endpoint definitions, prediction horizons, and validation settings.

## 6 Clinical applications of risk prediction models for diabetic macrovascular complications

Risk prediction models are clinically meaningful only when estimated risk can be translated into preventive, diagnostic, therapeutic, and follow-up strategies. In T2DM, these models have evolved from simple tools for cardiovascular risk classification toward instruments that may support treatment intensity, screening strategies, longitudinal monitoring, and EHR-based clinical decision support. Contemporary guidelines increasingly emphasize risk-stratified management, particularly in patients without established cardiovascular disease, in whom absolute risk estimation may help guide preventive strategies and shared decision-making (27, 28).

### 6.1 Risk identification and stratification

The most established application of cardiovascular risk prediction models is identifying patients at increased risk of future cardiovascular events. Traditional tools such as the Framingham Risk Score, UKPDS Risk Engine, ADVANCE, and RECODe, together with newer models such as SCORE2-Diabetes, allow patients to be stratified according to estimated absolute risk over a predefined period. SCORE2-Diabetes represents a contemporary example, estimating 10-year fatal and nonfatal cardiovascular risk in European patients with T2DM without previous CVD by combining conventional cardiovascular risk factors with diabetes-specific variables, including age at diagnosis, HbA1c, and eGFR (27). The model was developed using 229,460 patients and externally validated in 217,036 individuals, with regional recalibration across four European risk regions (27).

The 2023 ESC guidelines further incorporated SCORE2-Diabetes into cardiovascular risk assessment for patients with T2DM without established ASCVD or severe target-organ damage (28). However, cardiovascular risk is not static and may change with age, diabetes duration, kidney function, smoking, weight, blood pressure, lipid levels, and treatment exposure. Therefore, repeated risk assessment may be more informative than a single baseline estimate, particularly when major clinical or therapeutic changes occur.

### 6.2 Guiding preventive and therapeutic strategies

Risk prediction can also support the intensity of preventive and therapeutic interventions. In primary prevention, absolute cardiovascular risk may inform decisions regarding lipid-lowering intensity, blood-pressure management, weight control, smoking cessation, and other risk-factor interventions. The ESC framework links estimated cardiovascular risk with treatment intensity and shared decision-making in patients with diabetes (28).

Nevertheless, risk scores should support rather than replace clinical judgment. Patients with established ASCVD, HF, or CKD may already have clear indications for cardioprotective therapies independent of calculated risk. Thus, risk prediction may be particularly useful in patients without overt cardiovascular disease, where the appropriate intensity of preventive treatment is less obvious. Future models may further move beyond predicting event probability toward estimating individualized treatment benefit, including the expected absolute risk reduction associated with specific interventions.

### 6.3 Guiding screening and follow-up intensity

Risk estimates may also help determine the intensity of cardiovascular surveillance. Patients at higher predicted risk may require more frequent reassessment of blood pressure, lipid profile, kidney function, glycemic control, treatment adherence, and cardiovascular symptoms. In selected patients, risk estimates may also support decisions regarding additional testing, such as cardiac biomarkers, ankle–brachial index, carotid imaging, or coronary artery calcium assessment.

However, high predicted risk alone should not automatically trigger indiscriminate cardiovascular imaging or screening. Current guideline-based management emphasizes selective testing according to symptoms, clinical findings, and whether additional information is likely to change management (28). Therefore, risk models are most appropriately used as part of a stepwise evaluation strategy rather than as isolated thresholds for diagnostic testing.

### 6.4 Integration into EHR-based clinical decision support

Integration of prediction models into electronic health records represents an important pathway toward routine implementation. EHR-based systems can automatically retrieve demographic characteristics, laboratory values, diagnoses, medications, and other predictors, generate updated risk estimates, and link these results to evidence-based treatment recommendations.

Randomized studies have shown that cardiovascular clinical decision-support systems can improve risk-factor management. In a cluster-randomized trial involving 7,914 high-risk adults, an EHR-based decision-support system identified prioritized treatment opportunities for blood pressure, lipids, weight, smoking, and aspirin use and produced a more favorable change in predicted 10-year cardiovascular risk than usual care (125). More recent cluster-randomized evidence in community health centers similarly demonstrated the feasibility of real-time cardiovascular risk assessment combined with personalized, prioritized treatment recommendations, including patients with diabetes or established ASCVD (126).

However, successful implementation requires more than embedding an algorithm into an EHR. Data completeness, interoperability, alert fatigue, workflow burden, transparency, recalibration, and clinician acceptance can all influence real-world effectiveness. Therefore, future prediction systems should integrate automatic risk updating with interpretable and actionable recommendations and should be evaluated not only by discrimination and calibration, but also by whether their use changes clinical decisions, improves treatment implementation, and ultimately reduces cardiovascular events. This transition from Prediction to Decision to Benefit should represent the ultimate goal of clinical translation.

## 7 Major challenges facing risk prediction models for diabetic macrovascular complications

Although risk prediction models for diabetic macrovascular complications are increasingly evolving toward multimodal and artificial intelligence–based approaches, only a limited number of tools have been successfully implemented in clinical practice and shown to improve patient outcomes. The current challenges have therefore shifted from merely improving predictive performance to addressing the entire translational pathway, including study design, model generalizability, data quality, clinical decision support, and real-world implementation.

### 7.1 Lack of standardization in study design and outcome definitions

Substantial heterogeneity exists across current studies in terms of population selection, prediction start point, prediction horizon, and outcome definitions. Different models have been developed for the general population, individuals with diabetes, primary or secondary prevention settings, and short-term or long-term risk prediction. These models therefore address fundamentally different clinical questions, and their predictive performance cannot be compared directly. Previous studies have shown that some structurally simple traditional models may even outperform complex artificial intelligence models in independent cohorts, suggesting that greater model complexity does not necessarily translate into greater clinical value (127) Model performance is also influenced by the characteristics of the development population, regional event rates, and healthcare settings. Models such as QRISK3, ADVANCE, DIAL, and SCORE2-Diabetes were each developed for clearly defined target populations and clinical scenarios and generally require recalibration before they can provide reliable absolute risk estimates in different countries or cohorts (25, 27, 128–130). In addition, the use of different outcomes, including coronary heart disease, stroke, heart failure, cardiovascular death, and various composite endpoints, further reduces comparability across studies (131–133). Future studies should standardize target populations, prediction horizons, and outcome definitions, as well as harmonize variable collection and event adjudication procedures.

### 7.2 Insufficient external validation and limited generalizability across populations

Random data splitting, cross-validation, and bootstrap resampling can assess only the internal stability of a model and cannot substitute for independent external validation. As population characteristics, treatment patterns, and event rates change, both model discrimination and calibration may deteriorate, a problem that is particularly pronounced in machine learning models (133). Most existing models have been developed in European, North American, or single-center cohorts, with insufficient validation across countries, ethnic groups, and primary care settings (134–136). Models without independent validation should be regarded only as proof-of-concept tools. Before they are used for risk screening or treatment decision-making, multicenter external validation is essential (137, 138). Future studies should report discrimination, calibration, Brier scores, and decision-curve analysis simultaneously and should perform recalibration or model updating according to the characteristics of the target population.

### 7.3 Data quality, model bias, and inadequate fairness

Real-world data commonly contain missing values, coding errors, inconsistent measurement frequencies, and outcome misclassification. Complex algorithms may also learn proxy variables related to healthcare processes rather than true disease characteristic (139, 140). In addition, the relatively low incidence of macrovascular events and imbalanced sample sizes across subgroups may introduce class bias and reduce algorithmic fairness (133). Model performance is also affected by the study population, variable quality, event rate, and validation strategy. Consequently, C-statistics vary substantially across studies, and both traditional and artificial intelligence models exhibit considerable heterogeneity in performance (24, 99, 101, 127, 141–144). Comparisons between algorithms should therefore be conducted using the same dataset, the same predictor variables, and a standardized validation framework. Evaluation should extend beyond the AUC to include calibration, clinical net benefit, fairness, and implementation costs.

### 7.4 Limitations in dynamic prediction, model interpretability, and clinical actionability

Most existing models are still based on measurements obtained at a single time point and therefore cannot adequately capture how cardiovascular risk changes with diabetes duration, treatment, and metabolic control. Although time-varying predictors may improve the temporal relevance of a model, their incremental predictive value remains limited, and the usefulness of dynamic prediction depends on variable selection, measurement frequency, and modeling strategy (131, 145). In addition, current artificial intelligence models largely remain limited to explaining variable importance and generally cannot determine which risks are modifiable, when intervention should be initiated, or how intervention should be delivered. Future prediction models should therefore move beyond risk estimation toward clinical decision-support systems that integrate risk prediction with screening strategies, treatment selection, and follow-up management.

### 7.5 Limited clinical translation and real-world implementation

High predictive performance does not necessarily translate into clinical benefit. Most existing studies have stopped at model development and retrospective validation and lack prospective evidence demonstrating that model use can improve clinical decision-making, increase adherence to guideline-recommended care, reduce cardiovascular events, or achieve cost-effectiveness (146). With the widespread use of cardioprotective and renoprotective therapies, including sodium–glucose cotransporter 2 inhibitors and glucagon-like peptide-1 receptor agonists, the baseline risk of patients continues to change, requiring models to undergo continuous updating and recalibration (147, 148). In addition, most existing models focus on a single outcome, whereas myocardial infarction, stroke, heart failure, and death share multiple risk factors. Multi-task learning offers a new approach for jointly predicting these outcomes, although its clinical value requires further validation (149–151). Future research should shift its focus from asking whether a model is accurate to determining whether it changes clinical decisions and improves patient outcomes. Integration with electronic health records, continual learning, implementation science, and prospective randomized studies will be essential to complete the translational pathway from Prediction to Decision to Benefit (152).

## 8 Emerging prediction models and future directions: from risk identification to clinical decision-making

To address the limitations of current risk prediction models, including restricted data dimensions, static risk assessment, single-outcome prediction, and inadequate clinical translation, risk prediction for diabetic macrovascular complications is evolving from conventional risk scores toward intelligent prediction systems characterized by multimodal data integration, dynamic risk assessment, multi-outcome prediction, and treatment-oriented decision support. Future prediction models should not only estimate the probability of cardiovascular events but also answer clinically relevant questions, including which event is most likely to occur, how risk evolves over time, when intervention should be initiated, and which therapeutic strategy is most likely to achieve the greatest net clinical benefit (**Figure 2**).

**Figure 2:**
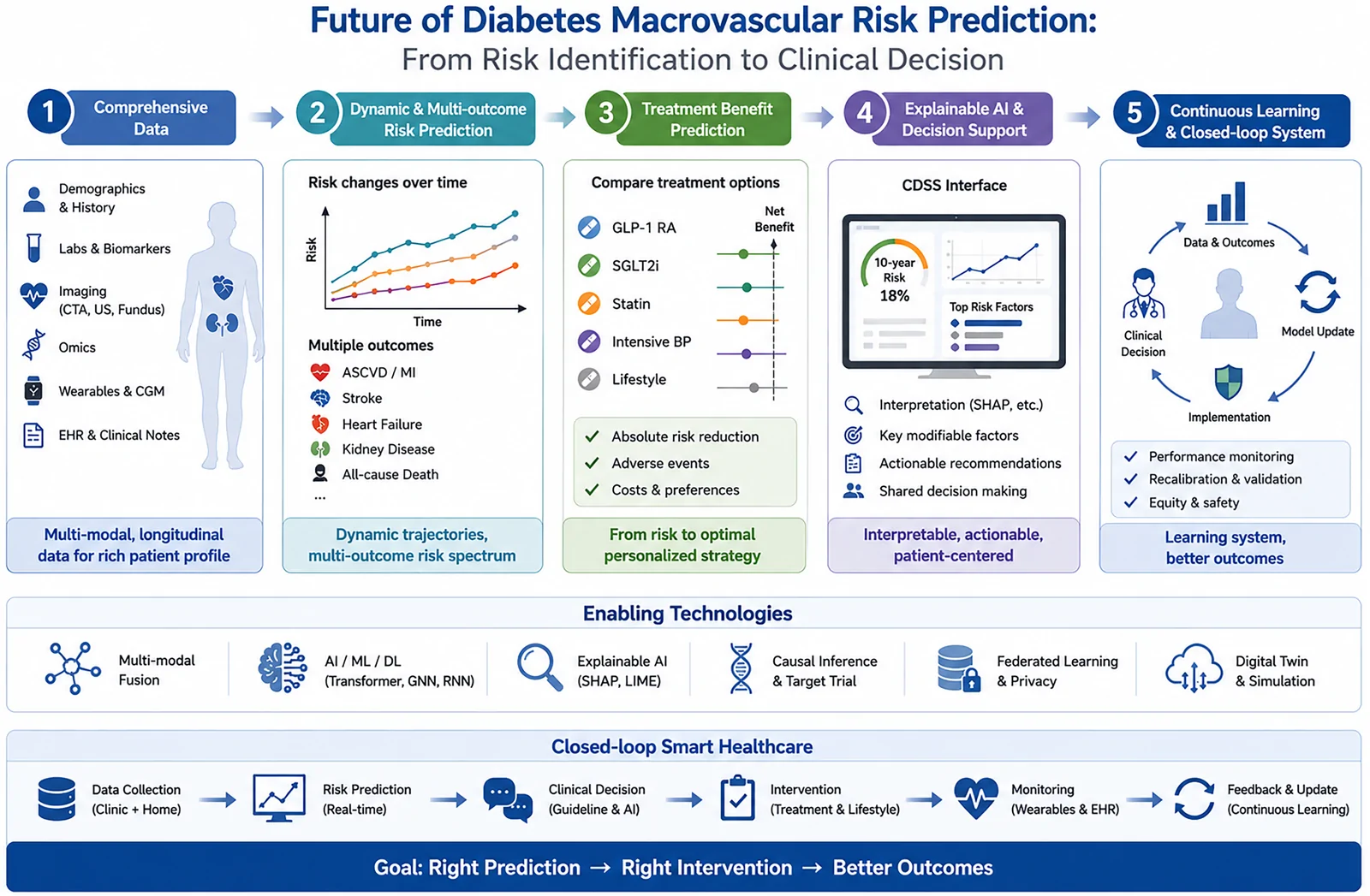
Future paradigm of risk prediction for diabetic macrovascular complications: from risk identification to intelligent clinical decision-making.

### 8.1 Multimodal, dynamic, and multi-outcome prediction

Future risk prediction will expand beyond conventional clinical variables to incorporate multimodal data derived from clinical characteristics, medical imaging, multi-omics technologies, EHRs, and wearable devices, thereby improving risk stratification through the integration of complementary information from different data modalities. Meanwhile, prediction strategies are expected to evolve from single baseline assessments to dynamic risk updating. Time-dependent Cox models, joint models, and Transformer-based architectures can integrate longitudinal clinical data to characterize individual risk trajectories over time (153, 154). Furthermore, multi-task learning, retinal imaging, and multimodal integration of conventional Western medicine and traditional Chinese medicine (TCM) are driving the transition from predicting a single cardiovascular endpoint to constructing comprehensive multi-outcome cardiovascular risk profiles (155–158). Future studies should focus on evaluating the true incremental value of newly incorporated data modalities with respect to model calibration, risk reclassification, clinical net benefit, and patient outcomes, rather than simply increasing the number of predictor variables (159).

### 8.2 From prognostic prediction to individualized treatment benefit prediction

Future prediction models should evolve beyond estimating disease prognosis to predicting individualized treatment benefit. By integrating disease phenotypes, genetic background, subclinical atherosclerosis, and treatment-related information, these models may estimate the absolute risk reduction and net clinical benefit associated with different therapeutic strategies (160–163). In addition to conventional performance metrics such as the AUC and model calibration, clinical utility should also be evaluated using decision-curve analysis and absolute risk reduction, thereby providing a more comprehensive assessment of model value in clinical practice (164, 165). Emerging analytical approaches, including causal inference, target trial emulation, and individualized treatment effect estimation, offer new methodological frameworks for precision treatment with glucagon-like peptide-1 receptor agonists (GLP-1RAs), sodium–glucose cotransporter-2 inhibitors (SGLT2is), statins, and other cardioprotective therapies. Nevertheless, their clinical utility requires further prospective validation (166–171).

### 8.3 Foundation models, digital twins, and intelligent simulation

Foundation models, large language models (LLMs), and digital twin technologies are driving the evolution of cardiovascular risk prediction from conventional structured-data analysis toward intelligent clinical simulation. LLMs can automatically extract clinically relevant information from electronic health records, facilitate risk interpretation, and support human–computer interaction. However, to ensure reliable prediction, they should be integrated with structured prediction models and evidence-based clinical rules rather than being used as standalone prediction tools (172–175). In parallel, digital twins enable the simultaneous integration of clinical data, medical imaging, multi-omics information, and wearable device data to dynamically simulate changes in cardiovascular risk under different therapeutic strategies. This capability facilitates the transition from risk prediction to clinical simulation and ultimately to decision intelligence (176, 177).

### 8.4 Continual learning and closed-loop intelligent healthcare

Future prediction models should establish hierarchical application frameworks tailored to different healthcare settings while incorporating continual learning capabilities. Emerging techniques, including federated learning, transfer learning, and continual learning, can facilitate multicenter collaboration, model transfer, and dynamic model updating, thereby enabling adaptation to newly introduced therapies and evolving disease patterns (178–181). Ultimately, prediction models will be seamlessly integrated into electronic health record systems and clinical decision support systems to establish an intelligent healthcare ecosystem encompassing data acquisition, risk prediction, result interpretation, treatment recommendation, therapeutic feedback, and continuous model updating, thereby forming a closed-loop framework for precision healthcare.

### 8.5 From predictive performance to patient benefit

The evaluation of future prediction models should shift its emphasis from predictive performance to clinical value. In addition to discrimination and calibration, greater attention should be paid to clinical net benefit, cost-effectiveness, physician workload, and patient-centered outcomes (182, 183). Risk prediction should directly inform decisions regarding the selection of imaging examinations, initiation of GLP-1 receptor agonists, SGLT2 inhibitors, and other cardioprotective therapies, as well as the development of individualized follow-up schedules and lifestyle management strategies (184–186). Future research should establish a complete translational pathway encompassing model development, external validation, continual updating, clinical decision-making, and patient benefit, supported by prospective impact studies and implementation science. Such efforts will be essential for enabling cardiovascular risk prediction models to achieve meaningful integration into routine clinical practice (187).

## 9 Conclusions

Risk prediction models for diabetic macrovascular complications have evolved from traditional statistical risk scores based on a limited number of conventional risk factors to artificial intelligence–driven models integrating electronic health records, medical imaging, multi-omics data, and wearable device data. Clinical characteristics and routine laboratory biomarkers remain the cornerstone of model development, whereas vascular imaging, molecular omics, and digital biomarkers provide novel sources of information for identifying subclinical vascular disease and characterizing dynamic cardiovascular risk. Traditional statistical models retain substantial advantages in interpretability and clinical applicability, while machine learning and deep learning have markedly enhanced the ability to analyze nonlinear, high-dimensional, and multimodal data. More recently, multi-task learning, foundation models, and digital twin technologies have further advanced cardiovascular risk prediction toward multi-outcome prediction, dynamic simulation, and intelligent clinical decision-making.

Despite these advances, existing prediction models continue to face several important challenges, including heterogeneity in study populations and outcome definitions, insufficient external validation, inadequate data quality, algorithmic bias, unfair comparison frameworks, limited clinical actionability, and a lack of robust evidence demonstrating real-world clinical benefit. Consequently, future research should move beyond the pursuit of higher predictive accuracy, as measured by metrics such as the AUC. Instead, greater emphasis should be placed on standardized study design, fair model comparison, model calibration, external transportability, clinical net benefit, cost-effectiveness, and patient-centered outcomes. Equally important, prediction results should be directly linked to diagnostic evaluation, therapeutic decision-making, and individualized follow-up strategies to maximize their clinical utility.

Ultimately, only by completing the transition from Risk Prediction to Clinical Decision and, ultimately, Patient Benefit can risk prediction models for diabetic macrovascular complications evolve from research tools into essential clinical infrastructure for precision prevention and individualized disease management.
